# Named entity linking of geospatial and host metadata in GenBank for advancing biomedical research

**DOI:** 10.1093/database/bax093

**Published:** 2017-12-28

**Authors:** Tasnia Tahsin, Davy Weissenbacher, Demetrius Jones-Shargani, Daniel Magee, Matteo Vaiente, Graciela Gonzalez, Matthew Scotch

**Affiliations:** 1 Department of Biomedical Informatics, Arizona State University, 13212 E Shea Blvd, Scottsdale, AZ 85259, USA; 2 Biodesign Center for Environmental Health Engineering, Arizona State University 781 E, Terrace Mall Tempe, AZ 85281 USA; 3 Institute of Biomedical Informatics, Perelman School of Medicine, University of Pennsylvania, 423 Guardian Drive, Philadelphia, PA 19104, USA

## Abstract

GenBank is a popular National Center for Biotechnology Information (NCBI) database for submission and analysis of DNA sequences for biomedical research. The resource is part of the Entrez environment which enables for cross-linking of concepts and entries in other participating NCBI databases such as Taxonomy, PubMed and Protein. For example, a GenBank record of an influenza A hemagglutinin gene DNA sequence might have a link to the Taxonomy database for the organism, a link to the related article in PubMed (if published) and a link to the Protein entry for the hemagglutinin protein. Despite its importance in biomedical research such as population genetics, phylogeography and public health surveillance, the host and geospatial metadata of genetic sequences in GenBank are not linked to any database. Therefore, to facilitate biomedical research based on georeferenced DNA sequences and/or DNA sequences with normalized host names, we designed and developed a framework that enriches GenBank entries by linking their host metadata to the NCBI Taxonomy database and their geospatial metadata to a comprehensive knowledge base of geographic locations called GeoNames. Here, we introduce a database created through the application of this framework to virus sequences in GenBank, and evaluate our normalization algorithms on a set of manually annotated records pertaining to viruses. Although currently applied to viruses, our framework can be easily extended to other organisms, and we discuss the potential utilization of our resource for biomedical research.

**Database URL**: https://zodo.asu.edu/zoophydb/

## Introduction

GenBank is a public database of nucleotide sequences developed and maintained by the National Center for Biotechnology Information (NCBI), which is part of the U.S. National Library of Medicine (NLM) of the National Institutes of Health (NIH) (1). With its participation in the International Nucleotide Sequence Database Collaboration (INSDC), NCBI exchanges sequences with international institutes such as the European Nucleotide Archive (ENA) (2) and the DNA Data Bank of Japan (DDBJ) (3). At the time of writing, GenBank contains a total of 200 877 884 sequences (4), along with pre-defined metadata describing each sequence. Over two million of these sequences are of virus origin, and include metadata such as the name of infected host of the virus, the location of infected host of the virus (LOIH) and the name of the gene the sequence corresponds to.

Viruses represent one of the principal causes of emerging and re-emerging infectious diseases across the world (5), and, therefore, understanding their evolutionary dynamics and geographical transmission, through diverse methods of analysis, is of critical importance. As one of the most comprehensive sources of virus sequence information, GenBank presents an invaluable resource for a wide range of virus-related research. It is frequently used in fields such as phylogenetics, phylogeography, molecular epidemiology, evolutionary biology and environmental health for studying viruses through a variety of different approaches. In addition to genetic sequence data, the rich metadata present in many GenBank records are vital for analysis and comparison. For instance, when mapping the global spread of each type of Dengue viruses across a time span of 70 years, Messina et al. extracted the type and geographical coordinates of 1070 GenBank records pertaining to Dengue viruses from their respective metadata fields (6). Similarly, Scotch et al. also utilized the geospatial metadata available for GenBank records when conducting a phylogeographic analysis of Influenza A H5N1 viruses isolated from Egypt (7).

One significant challenge faced by researchers in their efforts to incorporate GenBank metadata within their study, is the task of appropriately normalizing the data so that it is usable. Although GenBank contains distinct fields for storing sequence-related metadata, it does not place strict constraints on values that an author may enter for each field. As a result, many of the metadata fields in GenBank are semi-structured in nature, and must be processed before being utilized by a researcher. For instance, the host field of GenBank records with accession numbers AB618040 (8), AB618529 (9) and AJ312308 (10) contains the values ‘Homo sapiens’, ‘Homo sapiens 54-years-old female’ and ‘Man’, respectively, to denote the same species. Therefore, if a researcher intends to focus on virus sequences infecting, for example, humans and chimpanzees, they would first have to guess the different possible ways of denoting human and chimpanzee hosts, then query the GenBank website for each such possibility, and finally normalize each host field manually to allow grouping based on its value.

When extracting geographic metadata denoting the LOIH of a virus sequence, researchers may frequently have to perform an additional step of integrating geographic information from different fields in the GenBank record, prior to normalization. The designated field for storing the LOIH of sequences in GenBank is called the *country* field. Despite its name, the country field may contain geographic metadata of varying degrees of specificity, rather than only country-level information. For instance, the annotated data in the country field of the GenBank record with accession number CY045959 is ‘Canada: Ontario’ (11). Because of the specific nature of virus nomenclature, additional geographic information may often be found in the strain field and isolate field of GenBank records. For instance, the annotated data in the strain field of this record is ‘A/Toronto/T5294/2009(H1N1)’ (11). Combined with the information in the country field, it can be inferred that the LOIH of the virus is ‘Toronto, Ontario, Canada’. This process of extracting, integrating and normalizing the LOIH of sequences from GenBank record metadata can be highly challenging, especially when a researcher is not very familiar with the geographic region in which the study is being conducted. The ambiguous nature of many locations can make this process even more difficult. For instance, the location ‘Malang, Indonesia’ may be mapped to 20 distinct geo-coordinates based on GeoNames (12), a comprehensive database of geographic locations across the world. In 2005, GenBank introduced the lat_lon field (13) which, in the case of viruses, may be used to store the specific latitude and longitude coordinates of their LOIH. However, in our review, we found that this field is missing in over 99% of all GenBank records pertaining to viruses. Therefore, for the large majority of GenBank records, the task of geocoding is left to each individual researcher.

In this study, we describe the design and development of an integrated framework for normalizing host and location metadata in GenBank records pertaining to viruses. We applied a rule-based framework to map the name and location of the infected hosts of viruses to their corresponding NCBI taxonomy IDs (14) and GeoNames IDs (12) respectively. Our algorithm successfully linked 1 971 328 GenBank records to the GeoNames database, and 1 592 541 GenBank records to the Taxonomy database based on their host names. Prior to normalizing the LOIH of virus sequences in GenBank, we first used an automated approach to integrate data from different fields in the record which may contain geographic metadata. Therefore, our database includes the most comprehensive geographic metadata denoting the LOIH of each virus sequence in GenBank, which our algorithm is capable of extracting. To the best of our knowledge, this is the first framework that normalizes these two types of GenBank metadata for all virus-related GenBank records.

Given the significance of normalized GenBank metadata in a wide range of virus-related studies, our framework would help support a variety of different approaches used for understanding and/or analyzing virus epidemiology, migration patterns and evolutionary dynamics. This, in turn, may lead to major advances in infectious disease surveillance, and vaccine design and distribution, thereby enhancing our ability to control and contain disease outbreaks. In addition, our normalization algorithms linked each GenBank metadata to widely used and well-managed databases. This would facilitate cross-database queries, allowing the conduction of many new analytical studies. Moreover, the methods of normalization described here may also be easily applied to create similar databases for organisms such as bacteria or eukaryotes. Therefore, the work presented in this paper has the potential to considerably accelerate research in diverse biomedical fields.

### Related work

Over the past few years, NCBI has undertaken several large-scale efforts to add more structure to its data, resulting in the development of valuable resources such as BioSample (15, 16), Refseq (17, 18), NCBI Virus Variation (19, 20) and NCBI Viral Genomes (21, 22). These resources facilitate curation of GenBank metadata and are crucial for advancing biomedical research. However, we believe that our framework is distinctly different from each of them and serve a purpose not yet satisfied by any existing resource that we are aware of. The BioSample project represents a significant attempt by NCBI to integrate data across different resources, and provides an intuitive interface to facilitate submission of rich and consistent metadata. However, it relies on manual submission of metadata and is not linked to a large section of virus GenBank records. The Refseq database is a widely used resource within the research community which includes non-redundant, well-annotated genetic sequences but it requires manual curation of data, and, once again, a large portion of virus GenBank records do not have Refseq links. The NCBI Virus Variation project, which is part of the NCBI Viral Genomes project, utilizes a semi-automated pipeline for mapping GenBank metadata, including host and geographic metadata, to a controlled vocabulary. However, the pipeline is currently applied to newly-released GenBank records pertaining to seven viruses only. In contrast, we have successfully applied our automated system to over two million GenBank records pertaining to viruses. Moreover, the pipeline used by the NCBI Virus Variation project appears to map the geographic metadata of virus records to their corresponding countries/continents/regions only to allow recognition of up-to country-level hierarchy, while our framework normalizes the metadata to specific GeoNames entries (which includes their geographic coordinates) and is capable of recognizing up-to state/province-level hierarchy. Also, unlike our system, the NCBI Virus Variation pipeline appears to map host names to a controlled vocabulary of taxonomic host groups rather than specific taxonomy ids.

Although this work represents the first effort to create a comprehensive database including the normalized forms of the infected host and LOIH of all virus sequences in GenBank, several attempts have been previously made to normalize different GenBank metadata fields for different organisms. In our prior work (23), we used a rule-based approach similar to the one described here to extract, integrate, and normalize the LOIH of virus sequences in GenBank. However, instead of applying our approach to develop a database of virus-related GenBank records with normalized LOIH, we used it to develop a system for enhancing existing geographic metadata in ‘insufficient’ virus-related GenBank records by extracting additional information from linked full-text publications. We defined ‘Insufficiency’ as geographic metadata which was not more specific than Administrative Division 1 (ADM1) level, that is, state or province level. For instance, ‘Arizona, USA’ would be categorized ‘insufficient’ while ‘Maricopa County, Arizona, USA’ would be categorized ‘sufficient’. Therefore, once our system found ‘sufficient’ geographic metadata in a GenBank record, it would stop searching. For instance, if the geographic metadata in a record was ‘Tempe, Maricopa County, Arizona, USA’, our system would stop searching once it found ‘Maricopa County’, thereby missing the more specific location ‘Tempe’. Here, we updated our algorithm so that it finds the most specific geographic location, along with its parent ADM1 and country-level location, if present, for semantic context. Therefore, in the previous example, our current system would extract, and subsequently normalize, ‘Tempe, Arizona, USA’. Moreover, the rules for LOIH extraction in the system developed through our prior work were primarily designed for GenBank records pertaining to only the influenza virus. For this study, we added rules to optimize geographic metadata extraction for non-influenza viruses as well, and introduced additional features, such as a simple Lucene-based spell corrector, to minimize errors for all organisms. Furthermore, in our prior work, we used an SQL database for storing and querying the GeoNames knowledge base (KB). Here, we migrated to a Lucene index representation of the KB to enable faster queries.

In another recent work, Gratton et al. (13) utilized an automated approach for geocoding all previously un-geocoded GenBank records associated with tetrapods. However, they did not extend their study to include viruses, and limited the extraction of geographic metadata from the *country* field only, while we integrated geographic metadata from different fields in virus-related GenBank records for this study. Furthermore, they mapped the extracted geographic metadata to their respective latitude and longitude coordinates, while we mapped sequences to their corresponding GeoNames IDs, whenever possible, in addition to their geo-coordinates. This would enable cross-database studies involving the GeoNames database and the GenBank database, and provide a unique, normalized string representation of each LOIH to facilitate studies such as discrete phylogeography, where each LOIH is represented as a discrete character state (24).

Recent efforts have also been made to extract and normalize non-geographic metadata in GenBank. For instance, Sarkar (25) extracted the anatomical source of microbiome bacteria in ten mammalian hosts from the *isolation_source* and *note* fields in GenBank records, and normalized them using existing ontologies and annotation services available through the National Center for Biomedical Ontologies (NCBO) (26). In a separate work, Chen and Sarkar (27) conducted a feasibility study for normalizing the *host* and *isolation_source* fields in GenBank. They applied an automated approach to normalize *host* fields to their corresponding Taxonomy IDs, and used the NCBO web service annotator for normalizing the *isolation_source* field based on different ontologies. However, their work only involved an exploratory analysis of GenBank records, and no datasets including the normalized fields was made publicly available. Another related work by Sinclair et al. (28) introduced Seqenv, a software for linking genetic sequences to the Environmental Ontology (29). However, Seqenv takes genetic sequences as input instead of GenBank records, and the linking is performed based on the *isolation_source* field in GenBank. Therefore, it is specifically geared toward assisting researchers specializing in environmental genomics while our framework serves as a general framework for normalizing GenBank metadata, which may address the needs of diverse research areas.

Research in the emerging domain of *viroinformatics* has also lead to the development of many computational tools and databases to support the work of virologists. Sharma et al. (30) provided an exhaustive list of such resources, and included key features and functions of each resource. However, none of the listed resources was reported to have used computational methods for normalizing the host and geospatial metadata of all virus sequences in GenBank.

Outside of GenBank and viral genomics, different normalization methods have been utilized in a wide range of studies to normalize mentions found in free-text articles (31–35), tables and lists in web documents (36, 37), social media (38) and other databases/KBs (39). Although we exploited the basic principles involved in some of these normalization techniques, which are commonly used by researchers, the exact heuristics applied here remain unique to our study.

## Materials and methods

Our study can be divided into three distinct stages: (i) Database Design and Development, (ii) Entity Normalization, (iii) Evaluation. Below, we describe each stage in detail.

### Database design and development

#### Database design

**Figure 1.**
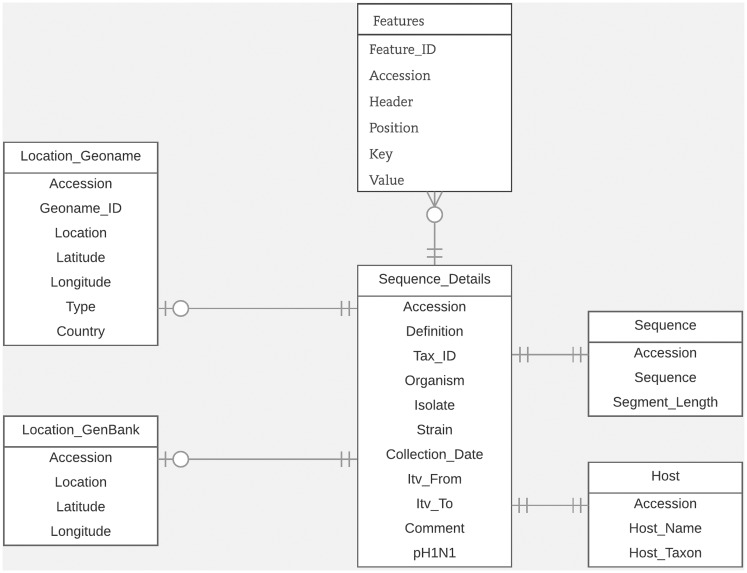
Database schema.

For this study, we designed an efficient and flexible database schema. In Figure 1, we illustrate the portion of the schema relevant to the task of entity linking. Within our database, the GenBank accession number is the main identifier used to connect all related metadata for each virus sequence. We organized the database around the ‘Sequence_details’ table which includes sequence metadata extracted from important fields in GenBank records such as the *organism* field, *isolate* field, *strain* field and *collection_date* field. We stored the data extracted from the *host* field, along with their normalized forms, in the ‘Host’ table. We stored the data from the *country* field, along with the latitude and longitude coordinates (which we derived from the *lat_lon* field), in the ‘Location_GenBank’ table. In the table ‘Location_Geoname’, we saved the integrated LOIH which we extracted from the relevant fields in each GenBank record. In this table, we also stored its corresponding GeoNames ID and latitude and longitude coordinates. We chose to use separate tables for storing the normalized host name and LOIH of each virus sequence to facilitate updating and/or analyzing the novel pieces of information derived through this study. We stored additional metadata from the ‘Features’ section of each GenBank record in the ‘Features’ table. Here, we utilized a flexible structure by using ‘Key’ and ‘Value’ columns to store each feature. Finally, we stored the entire nucleotide sequence included in each GenBank record in the ‘Sequence’ table.

#### GenBank data download

NCBI offers several web-based services to access or download the entire GenBank database. Here, we used the anonymous ftp server located at https://www.ncbi.nlm.nih.gov/genbank/ftp/ to acquire all GenBank records pertaining to viruses listed in the *gbvrl* files (excluding laboratory strains). Using a parser written in Java, we sequentially downloaded all GenBank flat files corresponding to virus nucleotide sequences from the anonymous ftp server. After downloading each file, we ran our parser to automatically extract relevant data for each sequence contained in the file, and stored them in our SQL database.

### Entity normalization

The task of normalization aims to map the mention of a concept to its corresponding ID in a predefined KB (40). For example, in the sentence ‘one SOR strain that was also isolated from a human in Germany’ (41), p. 2052, the mention ‘human’ can be linked to the concept Homo sapiens (ID: 9606) in the NCBI Taxonomy (42) and the mention ‘Germany’ to the concept of Federal Republic of Germany (ID: 2921044) in the GeoNames database (43). The normalization task is also known as ‘concept mapping’, ‘concept grounding’ or, as in our study, ‘entity linking’ when the concepts are only limited to entities. In entity linking, the mention of concepts, such as quality, process or events, is excluded from normalization.

Normalizing concepts in documents is made difficult due to the presence of various linguistic phenomenons. An intuitive approach to normalize the mention of a concept appearing in a document is to compare the mention with each entry in the chosen KB. If an entry matches exactly with the mention, the ID of the entry is linked to the mention. However, synonyms, polysemy, acronyms and spelling variations render a search by exact match ineffective (40).

When exploring the feasibility of normalizing concepts in the semi-structured *host* and *isolation_source* fields of the GenBank database, Chen et al. (27) noted that the *host* field often included the common names of the host, rather than their scientific names, in a wide range of different formats, along with additional information about the host, such as its age and gender (e.g. for accession CY138679 (44), the field *host* is ‘American black duck; gender M; age L—Local’). The *isolation_source* field presented an even richer syntactic and semantic diversity since its values varied based on the anatomy of the hosts. Therefore, in both cases, complex methods are required to successfully normalize the fields, and a simple search by exact match would likely be ineffective. Complex approaches of entity normalization rely on the exploitation of the properties of mentions and concepts, along with the contexts in which they appear (45). Below, we list some of the common features used in such complex approaches for entity normalization:

- **Names similarities:** The most intuitive and commonly used property to link a concept to a mention is the similarity between their names. When a strict string matching is not directly applicable, many named entity linking systems compute a distance of some sort, such as the Hamming distance or edit distance, between the string of the mention and the names of different concepts in the KB, to search for the closest concept. Some systems may choose to perform partial string matching instead of computing a string similarity score, while others may apply existing or domain-specific spell correctors to find the closest lexical and/or phonetic matches.
- **Concept popularities:** Some concepts are more frequently used than others. For example, if the name ‘Marie Currie’ is mentioned in a document, it is more likely to refer to the famous Polish physicist than the less famous American rock singer, ‘Marie Michelle Currie’. A simple metric to confirm this claim may be derived by comparing the number of Wikipedia articles referring to the physicist with the number of articles referring to the rock singer. An *apriori* probability can model this likelihood and be used to bias the default choice of a concept for a given mention.
- **Lexicalcontext:** When normalizing the mentions in a document, it may often be possible to exploit the lexical context around each mention (e.g. the words in the paragraph containing the mention) by comparing it with the lexical context of all possible concepts in the KB (e.g. the words describing the concept in the KB). The lexical context of the concept which corresponds to the mention is expected to be more similar to that of the mention in the document. However, when normalizing concepts in the fields of a database, such context may not always exist or be very informative. For instance, the host name entered in the GenBank record with accession KR349276 (46) is ‘mouse’. This mention is ambiguous with the taxonomy concepts Shrew mouse (ID: 10093) (47), House mouse (ID: 10090) (48) and Western Wild Mouse (ID: 10096) (49). However, it is not possible to exploit lexical context to disambiguate this mention since it is not surrounded by any other word in this field.
- **Semanticcontext:** The concepts discovered in a document are rarely independent of each other, and the chosen concept for a mention should be coherent with the concepts chosen for other mentions in the document. In our previous example, if the name ‘Marie Currie’ is found in a document mentioning the names ‘Cherie Currie’ and ‘Steve Lukather’, which match the names of the American rock singer Marie Michelle Currie's sister and husband respectively, it is more likely to refer to the American rock singer rather than the more famous Polish physicist. Therefore, the semantic context of a mention may often be used to successfully disambiguate the entities in a document.

In this study, we used several of the entity linking strategies listed above for normalizing GenBank metadata, in addition to using search by exact match. When normalizing the *host* field, we exploited the name similarities between mentions and concepts (through partial string matching based on the head of the mention phrase) as well as the popularity of the concepts. In case of geospatial metadata normalization, we exploited the semantic context of the mentions along with the name similarity (based on a domain-specific spell corrector which uses edit distance and phonetic similarity between strings to find matches) and concept popularity features. Geographic locations are hierarchical in nature and GenBank metadata often includes hierarchical information for the LOIH, which we refer to here as semantic context. For instance, if the ‘country’ field of a GenBank record contains ‘Paris, Texas, USA’, our algorithm would use ‘Texas’ and ‘USA’ as semantic context when disambiguating ‘Paris’. As a result, ‘Paris’, in this specific case, would be mapped to the GeoNames ID of 4717560 (50), representing the city of Paris in Texas, USA, rather than the GeoNames ID of 2988507 (51) representing the capital city of France, which is the more widely known of the two locations. Without taking the semantic context of ‘Texas, USA’ into consideration, our algorithm would have mapped Paris to the capital city of France. We did not utilize lexical context in either of the two normalization algorithms presented here since the description included in GenBank for each metadata is too short to benefit from this normalization strategy. Further details about each normalization method are described below.

#### Host normalization

**Figure 2.**
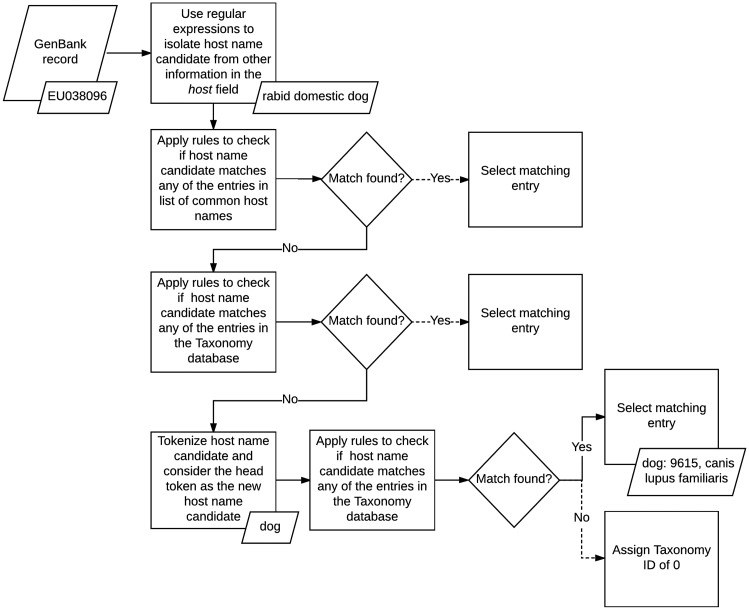
Host metadata extraction and normalization algorithm.

We normalized the host field in GenBank records by applying a set of matching rules in sequence (see Figure 2). First, we isolated the name of the host from any additional information the field may contain using a series of handwritten regular expressions. The regular expressions we applied were designed to recognize several formats followed by authors when entering this field during the sequence submission process. For example, based on one of our rules, we discarded any text in the field which followed the occurrence of the first punctuation mark, if the punctuation was not a period, and kept only the remaining phrase. For example, in GenBank record KT390491: ‘Abelmoschus angulosus; IC-140156’ (52), we only kept *Abelmoschus angulosus*.

Once we isolated the names of the hosts, we applied a set of rules to map the mention of common host names, such as mouse and human, to their corresponding IDs in the Taxonomy database. If none of these rules matched, then we implemented a second set of rules to search for regular patterns against the entire taxonomy tree, instead of a small set of common hosts. If no matching host name was found, we tokenized the name and checked if the head token matched any of the rules included in the second set. If a match was still not found, then we assigned the *host* field a Taxonomy ID of 0, to indicate that the host name of the record was unknown. At its current state, our host metadata normalization method does not utilize any spell corrector or string similarity scoring algorithm to find additional matches.

Although several NLP tools currently exist for the generic task of species normalization (34, 53), we opted to develop our own algorithm for this domain instead of adopting one of the existing tools. Most GenBank records pertaining to viruses contain very short descriptions of the infected host within the host field. In many cases, the included host name is a scientific name which can be directly mapped to an NCBI Taxonomy entry. Non-scientific host names used typically fall within a limited set of common host names. Therefore, we attempted to use a simple rule-based approach for normalizing the host names in GenBank records rather than applying more complex NLP tools. This allowed us to keep our methods as simple and efficient as possible while still having complete flexibility to make any changes needed to enhance performance specifically for this domain.

#### Geospatial metadata normalization

To extract and normalize the geospatial metadata of virus sequences in GenBank, we constructed a Lucene index of geographic locations, based on the GeoNames database, to serve as our KB of location names. The GeoNames database, which encodes the properties and hierarchical structure of over 10 million geographic locations, is a widely-used resource for geographic information extraction. However, it contains many entries such as ‘rat’ and ‘fox’ which may generate many false positives. Therefore, we collected different lists of commonly used words from different sources to filter them out. This includes a list of the names of common virus hosts and a list of English stop words (23). GeoNames also includes the alternate names of each location in different languages. We included these alternate names in our KB for all ADM1-level locations to maintain a high recall. For country-level locations we manually added commonly used country names and considered the Socrata dataset (54), which includes geospatial data for 243 countries, when adding these alternate names (23). For all other locations, we did not include any alternate name to minimize false positives. The choice of whether to add the alternate names in each case was based on a preliminary analysis we performed on a small set of records to determine the ideal configuration for minimizing false positives and false negatives.

**Figure 3.**
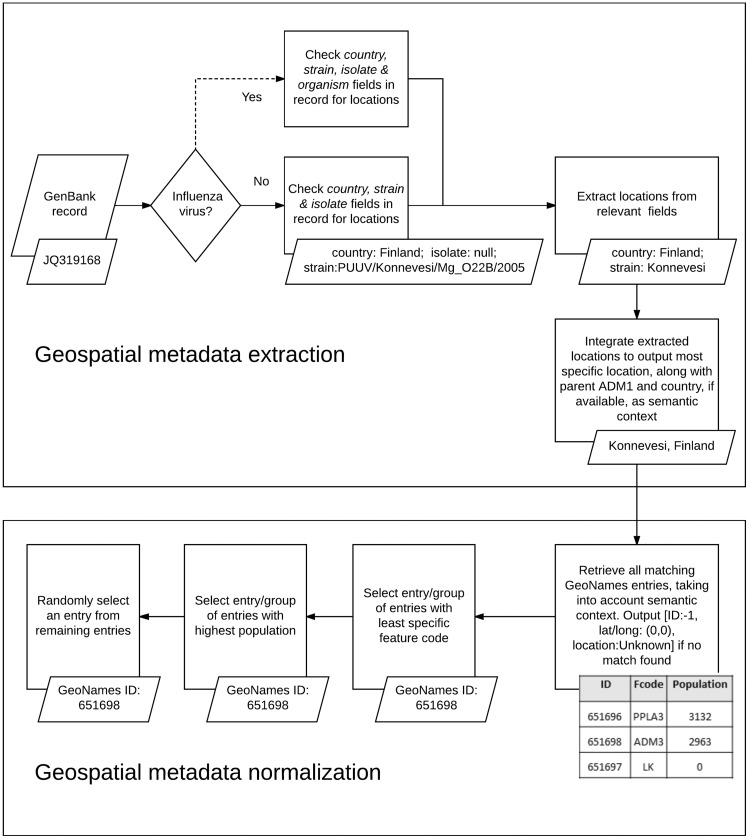
Geospatial metadata extraction and normalization.

We used the developed KB, along with a set of rule-based heuristics, to automatically extract, integrate and normalize geospatial metadata from multiple fields in virus-related GenBank records (see Figure 3). We analyzed the following GenBank metadata fields of all virus sequences: *country*, *strain* and *isolate*. As mentioned earlier, the *country* field is the designated field in GenBank for storing information about the LOIH of virus sequences but additional geographic information may often be found in the *strain* field or the *isolate* field of GenBank records. In case of GenBank records pertaining to influenza viruses, the strain name of the virus sequence may also be recorded in the *organism* field of the record, which, therefore, presents another potential source of information for the LOIH of the virus, especially when the *strain* field is empty. However, many species of viruses, such as the Puumala virus, contain location mentions (in this case Puumala) within their species name which do not refer to the LOIH of the specific virus sequence. Therefore, to avoid the possibility of including erroneous locations, and for simplicity, our system only analyzed the *organism* field for influenza viruses.

For each GenBank record included in our database, our system first segmented the string in each pertinent field of the record based on simple delimiters, and considered each segment to be a possible candidate location. It then searched our developed KB to find possible matches for each candidate location. In case of overlapping locations, it chose the location with the greater number of tokens. For instance, if the content of the *country* field in a GenBank record is ‘Sierra Leone’, our system would extract ‘Sierra Leone’ (55) as a single location although GeoNames includes separate locations named ‘Sierra’ (56) and ‘Leone’ (44) respectively. To avoid false positives, our system discarded any candidate location which consisted of only three letters, unless it corresponded to a US state postal code (e.g. NY for New York). If no match was found, our system removed words such as ‘state’, ‘county’, ‘region’, ‘east’ and ‘west’ from the candidate location name and re-initiated the search. If still no match was found, our system applied a simple Lucene-based spell corrector to check for misspellings. The spell corrector first checked if a match could be found by inserting a space after each character in the query string to handle cases like ‘NewYork’. If no match was found, it retrieved the top ten Lucene matches within two edit distance of the string if its length was greater than seven characters, and within one edit distance of the string if its length was greater than five characters but less than seven characters. The thresholds for choosing the edit distance limits were determined based on observations we made when analyzing system errors on a few preliminary datasets, separate from the one used in this study. Among the ten Lucene matches retrieved through this method, our spell corrector prioritized matches with the same phonetic representation as that of the query string (via the phonetic algorithm in Double Metaphone (57)) over those that did not have the same representation, and selected the top ranked match as the corrected spelling. It then integrated extracted location mentions to produce a coherent set of locations.

Our location integration algorithm functioned under the assumption that country-level locations are more likely to have been extracted correctly by our system than ADM1-level locations, which in turn are more likely to have been extracted correctly than locations less specific than ADM1. For instance, if our system extracted the locations ‘Grebe’ and ‘Russia’, it would disregard ‘Grebe’ since, according to the GeoNames KB, there is no location called ‘Grebe’ in Russia, and so it would assume that ‘Grebe’ was extracted incorrectly. Similarly, if it extracted the locations ‘Grebe’, ‘California’ and ‘USA’, it would once again disregard the location ‘Grebe’, even though one exists in the state of Oregon in USA, since none can be found in California, USA. Before integrating any location more specific than ADM1-level, we ensured that it was contained within any ADM1-level or country-level location extracted from GenBank. We did not control the coherence between locations beyond the ADM1-level since we considered hierarchical data in GeoNames to be adequately complete up to ADM1 level. For instance, we are confident that the GeoNames KB would include the parent country name and ADM1-level location name for all locations named ‘Grebe’. Therefore, our coherence checking process is more likely to lower false positives, than introduce false negatives. However, we are not as confident that GeoNames would correctly include the parent ADM2-level location and beyond for all locations, and so we chose not to check coherence beyond ADM1-level, to minimize the risk of missing valid locations. If multiple sets of coherent locations were found, our algorithm chose the set that provided more information. For instance, if our system extracted ‘Connecticut, USA’, and ‘Summit, New Jersey, USA’, it would choose the latter since that includes a larger set of coherent locations. After selecting a coherent set of locations, our algorithm outputted the most specific location in the set, along with its parent country and ADM1-level location, if available. For instance, if the selected set included ‘Chicago’, ‘Illinois’ and ‘USA’, our system would produce the integrated metadata ‘Chicago, Illinois, USA’. We included the parent country and ADM1-level locations to provide semantic context for our normalization algorithm, as we detail next.

When performing normalization, our system first searched our KB of geographic locations to retrieve all possible GeoNames entries for the location, using the most comprehensive information available. For instance, if the integrated geographic metadata was ‘Chicago, Illinois, USA’, a search was performed to retrieve all matches for ‘Chicago’ in the state of Illinois in the country of USA and, thus, the locations ‘Illinois’ and ‘USA’ were used as semantic context by the normalization algorithm. Next, it narrowed the search results to the group of entries which possessed the least specific feature code (code in GeoNames denoting the type of the location, e.g. country, state, city, etc.). For instance, in GeoNames, ‘Arizona’ can be both a state in USA (with a feature code of ADM1) and a populated place in the state of Texas, USA (with a feature code of PPL) but our system would only select the former entry since it has a less specific feature code. This heuristic is based on the assumption that the less specific a location is, the more widely known it tends to be, and authors typically tend to refer to more widely known locations (23). Our system then further narrowed down the search to the set of entries with the highest population. This heuristic is based on the assumption that geographic locations that have higher populations tend to be referenced more often by authors (23, 32). If the system still failed to uniquely identify the location, it randomly selected one of the possible entries. In case of records for which our system was unable to extract any location from any of the fields analyzed, the LOIH was listed as ‘Unknown’ with a GeoNames ID of ‘−1’, and the latitude and longitude fields were populated with ‘0’.

As we outline in *Related Work*, our geospatial metadata extraction and normalization algorithm expands upon our prior work (23) in this area. However, we made a significant number of changes to the pipeline to enhance its efficiency and accuracy, so that it may be more easily applied for the large-scale project undertaken here. Important updates include the following: (i) migration from MySQL database to Lucene index for storing the KB of geographic locations in order to enable faster queries, (ii) addition of rules to parse *strain* and *isolate* fields of non-influenza viruses, which tend to be less structured than those of influenza viruses, (iii) addition of a simple spell-corrector to account for spelling errors in GenBank, (iv) addition of rules to allow the system to extract the most specific LOIH of the virus, instead of simply extracting any location more specific than ADM1-level (e.g. if the complete geospatial metadata was ‘Chicago, Cook County, Illinois, USA’, our prior algorithm may not extract ‘Chicago’ since it would stop searching once it found ‘Cook County’) and (v) normalization to GeoNames IDs rather than latitude and longitude coordinates (this was a not a significant change with respect to implementation, but has important implications in supporting GenBank-related research by enabling cross-database queries).

### Evaluation

To evaluate the accuracy of our normalization algorithms, we randomly selected 100 GenBank accession numbers among those included within our database, for manual annotation. Two annotators, whose biomedical specialties required them to work extensively with GenBank records pertaining to viruses, annotated and normalized the LOIH and infected host of each selected GenBank record. In both cases, the annotators used the GenBank website to acquire pertinent GenBank metadata for completing their annotation tasks. For each virus sequence, they tried to find the most comprehensive information available in GenBank concerning its LOIH and host name, using all fields present in GenBank, regardless of if our program used the field. In addition, in case of host metadata, our annotators also used domain knowledge to annotate host names even when they were not included in GenBank. For instance, they automatically assigned ‘host = human’ to any HIV record. Also, the strain name of the influenza virus typically includes the name of its infected host, unless the host is human. Therefore, with the absence of any host metadata included in the strain field, it is reasonable to infer that the host was human for any non-laboratory strains.

After retrieving each relevant GenBank metadata, our annotators normalized it based on the selected KB. For normalizing geospatial metadata, they searched each location in the GeoNames website (12) to retrieve their corresponding GeoNames ID. Like our program, when multiple GenBank entries were available for a given location, they selected the one with the least specific feature code. For normalizing host names, our annotators used the NCBI Taxonomy website (58) to determine the Taxonomy ID of the host. In cases where they were unable to link a GenBank metadata to the selected KB, they inserted ‘0’ in the ID field.

Once the annotations were complete, we computed percentage agreement between our annotators for each annotation type to serve as a measure of inter-rater reliability. We chose to use percentage agreement, rather than Kappa statistic, because the number of possible categories in each annotation is over a million. The Kappa statistic is used to take into account agreement by chance (59). However, given the number of possible categories in each annotation, the possibility of agreement by chance is negligible. Other studies in information retrieval have used *f*-score as a measure of agreement (23, 60). However, in this study, each annotation simply involves entering a single value; therefore, the calculation of *f*-score would be redundant and a simple percentage agreement calculation is justified.

Once we completed the calculation of percentage agreement between our two annotators, a third annotator went through each case where they differed and selected the correct annotation to create our gold standard dataset. If it was unclear which annotation should be chosen, all annotators discussed the reason for the difference in annotation and mutually decided on one. Once the gold standard was created, we compared the annotations in the gold standard with the corresponding content in our database for measuring accuracy. In addition, to obtain a baseline performance measure for the host normalization task, we also computed the accuracy of the MetaMap (53) tool for this task on our gold standard dataset. When running MetaMap, we restricted sources to the NCBI Taxonomy vocabulary and retrieved the UMLS concept id with the highest score in each case. We then used the UMLS MRCONSO.RRF file to map the concept ids to their corresponding NCBI Taxonomy IDs. We applied the bias-corrected and accelerated (BCa) bootstrap method (61) with 10 000 iterations using the ‘boot’ package (62) in *R* to calculate the 95% confidence intervals (CI) for each accuracy value.

## Results and discussion

### Database statistics

**Figure 4.**
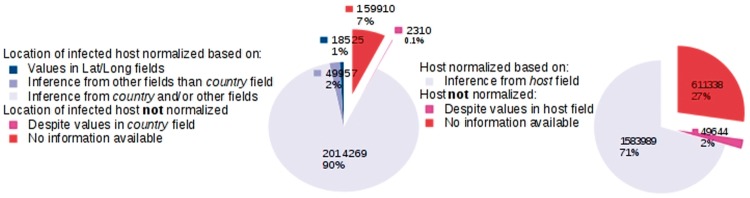
Database statistics.

We provide key statistics pertaining to our database, which currently contains 2 244 971 GenBank records corresponding to 162 043 distinct virus organisms (see Figure 4). We successfully mapped:

1. The LOIH of 2 014 269 (89.7%) records to their respective GeoNames IDs by our LOIH normalization algorithm. Only 18 525 (0.8%) of these records originally had values in their ‘lat_lon’ field.
2. The infected hosts of 1 583 989 (70.6%) records to their respective NCBI Taxonomy ID by our host normalization algorithm. None of the GenBank records contained a formal link between the host field and an entry in the NCBI Taxonomy database.

#### Host normalization analysis

Rule-based methods are known to fail to capture the infinite variety of the human language, and consequently, our approach is expected to be imperfect. The host names of 29.4% of the GenBank records in our database were not normalized and were assigned the Taxonomy ID of 0. 27.4% were not normalized simply because the value in the *host* field was left empty. However, for 2.2%, 49 644 instances with instances repeated corresponding to 6803 unique instances, the *host* field contained a value but our rules failed to find the corresponding Taxonomy ID. We randomly selected 100 unique instances from this set and analyzed the reasons for the failure of the rules. For 41 instances, the presence of an abbreviation made the exact matching impossible, for example, *C.tantalus* didn’t match with *Chlorocebustantalus* (ID: 60712) (63), and *tantalus* alone is not a concept in the taxonomy. For 35 instances, the *host* field contained the host name but also included additional information which was often not separated by delimiters from the host name, making the search difficult, for example, *marine Heterobranchia species* where *Heterobranchia* is found in the taxonomy (ID: 216305) (64) but the presence of *marine* and *species* leads our algorithm to fail. The last 24 instances were not found in the taxonomy due to misspellings like *Lepus europeaus* for *Lepus europaeus* (ID: 9983) (65), and missing entries in the Taxonomy database for alternative names of species such as *isard* for *Pyrenean chamois* (ID: 72545) (66) or species such as *Paradoxurus musangus*. These reasons can also be found together in the same name of host making its normalization even more difficult. Further research is needed to design dedicated strategies to discover the reasons for the failure of the rules and finalize the normalization.

#### Geospatial metadata normalization analysis

In case of geospatial metadata normalization, our system analyzed data from multiple fields in each GenBank record, and when running the pipeline, we recorded the number of locations extracted from each field. We found that our system extracted a total of 2 968 570 locations from the GenBank record fields analyzed. This count simply represents all unique locations extracted by our system per record, and includes duplicate locations, in cases where they were extracted from different records. For instance, given a sample of two records, one having the location ‘Chicago’ in the *strain* field and ‘USA’ in the *country* field, and the other having the location ‘USA’ in both the *strain* and *country* fields, the total number of extracted locations, counted through this method, would be three. The percentage of the 2 968 570 locations that were extracted from the *country*, *strain*, o*rganism* and *isolate* fields was 87.3% (2 594 402 locations), 17.3% (514 282 locations), 14.7% (434 931 locations) and 4.75% (141 064) respectively (the percentages do not add up to one since many of the locations were extracted from multiple fields, for example, if a location in a given record was collected from both the *strain* and *country* fields, it would be included in the count for both fields). Therefore, 12.7% of the locations were extracted from GenBank fields other than the *country* field. This indicates the importance of analyzing GenBank fields other than the *country* field for extracting geospatial metadata.

A comparison of the percentage of GenBank records in our database having missing values in the *country* field, with the percentage our algorithm failed to normalize, also illustrates the significance of integrating geospatial metadata from multiple GenBank record fields rather than only the *country* field. In 12.4% (278 350 records) of all GenBank records in our database, we did not find any data in the *country* field. However, we were unable to normalize the geospatial metadata of only 10.3% of the GenBank records in our database. This means that for at least 2.1% of the GenBank records, we added additional information from GenBank record fields other than the *country* field.

To obtain an estimate of the frequency with which our algorithm failed to extract geospatial data from the *country* field, we counted the number of records our algorithm failed to normalize despite the presence of geographic information in the *country* field. For a total of 2310 records (0.1% of all records), representing 13 unique LOIH and 14 unique locations, the *country* field contained geospatial metadata which our algorithm was unable to extract. Of the 14 locations missed, seven were missed because we did not include the alternate names of all locations from GeoNames. For instance, GeoNames lists ‘British Guiana’ as an alternate name for the main entry ‘Guyana’, but since we are not analyzing alternate names, our system failed to extract it. For four of the missed locations (e.g. ‘Kpokhankro’), we did not find any match in the GeoNames website when we manually searched for them. Therefore, the locations are most likely missing in GeoNames. Two of the locations were missed due to the presence of the word ‘the’ before the geographic location mention, for example, ‘The Netherlands’. Although, as described in *Methods*, we used a list of stopwords to remove every GeoNames entry from our database which was an exact match for one of the stopwords in our list (such as ‘but’), we did not remove stop words from within GeoNames entries which were composed of multiple words. For instance, we did not remove the string ‘but’ from within the GeoNames entry called ‘Ban Nong Yai But’ (67), which represents a city/town in Thailand. Similarly, we did not remove stop words from within strings extracted from GenBank metadata such as ‘The Netherlands’. In case of locations for which our algorithm failed to find a match in GeoNames, it removed words such as ‘state’, ‘county’ and ‘south’ and attempted to find a match again. However, stopwords such as ‘the’ were not included in this list since their presence may possibly provide valuable context (as in the case of ‘But’ in ‘‘Ban Nong Yai But’). Moreover, our spell correction algorithm only searched for matches within 1 or 2 edit distance of the candidate string, depending on the string length. Addition of ‘the’ represents an insertion of four additional characters (including space) and, therefore, our spell correction algorithm failed to find a match as well. The remaining location was missed due to the failure our algorithm to correctly identify the abbreviation ‘USSR’ standing for the former Union of Soviet Socialist Republic, now dissolved.

### Annotation statistics

Our gold standard annotation dataset includes 100 GenBank records with 64 distinct LOIH and 20 distinct host names. The percent agreement between our annotators for host and LOIH normalization was 95 and 83 respectively (Table 1). Differences in host annotation resulted from either of the two annotators missing a host name present in GenBank, erroneously adding a host name not present, not selecting the most specific host name available (e.g. deer instead of roe deer) or not annotating the host name of a record with missing host metadata even when it could be inferred based on the virus organism. In case of geospatial metadata annotation, our annotators annotated the same location in seven of the 17 instances where their final ID annotation differed. However, they disambiguated the locations differently. The remaining differences arose from missed locations.

**Table 1.** Inter-rater agreement and accuracy of normalization tasks based on manually created gold standard of 100 GenBank records

Task	Inter-rater agreement (%)	System accuracy (%)
Host metadata normalization	95	70
LOIH metadata normalization	83	87

### Accuracy statistics

We found that the accuracy of our normalization algorithms for host and geospatial metadata to be 70% (95% CI [0.60–0.77]) and 87% (95% CI [0.78–0.92]) respectively when evaluated on the manually annotated gold standard (Table 1). The baseline performance for host normalization using MetaMap was found to be 63% (95% CI [0.52–0.71]).

Of the 30 errors in host normalization, 28 were from lack of domain knowledge, 27 of which were specifically the result of the program not knowing that certain viruses affected only a single species of organism. One error resulted from the inability of the system to extract the host ‘bar-headed goose’ and in case of the remaining error, the program correctly extracted the host name but incorrectly normalized it to the ID of its parent organism.

MetaMap’s accuracy was found to be 7% lower than that of our system. As expected, MetaMap missed all host names where domain knowledge was required. In addition, in many cases it mapped the entities to higher level concepts than what was annotated by our annotators. For instance, MetaMap normalized the host ‘duck’ to the concept id corresponding to the genus ‘Anas’(68) while our annotators normalized it to the taxonomy id corresponding to the specific species ‘Anas platyrhynchos’ (69).

Of the 13 errors in LOIH geospatial metadata normalization, eight were due to disambiguation errors (same string representation of locations but different GeoNames IDs), three were due to missed locations, and the remaining two were due to the detection of locations not annotated by our annotators. The disambiguation errors resulted from the inability of our algorithm to choose the correct location based on exact string match. For instance, based on our annotation guidelines, the annotators normalized the location ‘Ningbo, Zhejiang, China’ to the GeoNames ID ‘1799395’ (70) which corresponds to the second order administrative division (ADM2) named ‘Ningbo Shi’ in GeoNames. Since ‘Ningbo Shi’ is not an exact match for ‘Ningbo’, our program incorrectly normalized it to the GeoNames ID ‘1799397’ (71) instead, which corresponds to the capital city of Ningbo Shi, and is named ‘Ningbo’ in GeoNames. Among the missed location errors, one was a result of GeoNames including a different spell variant of the location, which our system was not able to recognize. The remaining locations were missed because they were annotated based on the *title* field in GenBank (a field containing the title of a publication linked to the record) but our program does not extract metadata from the *title* field. Both of the locations extracted by our program but not annotated in our gold standard were valid locations. We chose to not include one of them in our gold standard because it was too ambiguous and it was not possible to correctly normalize it based on available information. The other was most likely missed by both of our annotators.

## Conclusion

In this study, we developed an automated framework for extracting and normalizing two different types of GenBank metadata which are widely used in different domains of biomedical research. We applied our framework to retrieve the host and geospatial metadata of over two million GenBank records pertaining to viruses, and link them to the NCBI Taxonomy database and the GeoNames database respectively. We have made the database including the normalized metadata publicly available to allow researchers to easily integrate them within their works and help accelerate biomedical discovery. In addition, we also created a manually annotated gold standard dataset consisting of 100 randomly selected GenBank records for evaluating the normalization algorithms. The percent agreement between our annotators was over 80% for the annotation of the two GenBank metadata types, which is adequately high. It was higher (95%) for host annotation than for geospatial metadata annotation (83%), illustrating the latter to be the more challenging of the two tasks when performed manually.

When evaluated on the gold standard set, our host and LOIH normalization algorithms achieved accuracies of 70% (95% CI [0.60–0.77]) and 87% (95% CI [0.78–0.92]) respectively. The majority of the errors in host normalization resulted from lack of domain knowledge, indicating the need to incorporate additional rules within our system to account for cases where a virus organism may only infect a single type of host organism. However, our current lack of such rules should not in any way reduce the applicability of our released dataset, since researchers are more likely to utilize it for GenBank records where the host name is not definitively known based on the nature of the virus organism. Our system correctly normalized the host name in all but two records, where specific domain knowledge was not required. In case of geospatial metadata normalization, the accuracy of our system was in fact higher than the inter-rater agreement calculated for its annotation. The systematic nature of our algorithm made it more suitable for this difficult task which requires extensive efforts when done manually.

Our gold standard dataset is relatively small and, since it was randomly selected, it often included duplicates of the most common hosts or geographic locations included in GenBank, leading to an even lower number of distinct metadata annotations. This is especially true for host metadata annotation, since the infected host in most records included within our dataset was ‘human’. Therefore, our measured performance may not be reflective of the average performance of the algorithms in other datasets.

In addition to evaluating our normalization algorithms on the gold standard dataset, we also performed supplementary analysis to investigate the completeness of the normalized host and geospatial metadata in our database. Our analysis showed that our normalization algorithms could normalize nearly all GenBank records for which the *host* and *country* fields were not empty. Although this does not necessarily mean that the extraction and normalization of metadata was performed correctly in each case, it nevertheless provides a simple measure of our system’s ability to extract metadata whenever available. In addition, our analysis also revealed the importance of including geospatial metadata from different GenBank record fields rather than only the *country* field.

Although various large-scale efforts are currently being made by NCBI to facilitate curation of rich and consistent GenBank metadata, we believe that the framework and database presented in this manuscript would continue to remain highly useful to researchers, both in the present time and in the near future. Manual annotation of millions of GenBank records could take years and is an expensive process. In contrast, our normalization algorithms take only a few seconds to process each record and could help considerably accelerate this process. Moreover, it also provides a standardized method for disambiguating metadata and may even help correct some errors made by humans. We have already applied our framework to create a comprehensive database including the normalized host and geospatial metadata of over two million GenBank records, which is easily accessible online. Our database has the potential to support a wide range of large-scale analyses involving viruses and would greatly benefit researchers working with virus GenBank records. Furthermore, by providing a thorough description and analysis of the geospatial and species metadata normalization methods we developed through our project, we hope to assist researchers working with similar normalization problems in any field.

We have made the source code for our framework available through github and it can be easily extended to other pathogens as well. The *country* and *host* field of all entries in GenBank are similarly formatted, regardless of which organism it pertains to. Therefore, it should be possible to use our framework, as it is, for extracting metadata from these fields for any pathogen. However, unlike viruses, most pathogens do not contain additional information pertaining to their location of collection in the other GenBank record fields analyzed by our algorithm, such as the *strain* field and *organism* field. Therefore, the inclusion of such fields would be unnecessary for other pathogens.

As future work, we plan to evaluate our existing algorithms on larger datasets, and work on improving their accuracy by including additional features such as a more sophisticated spell corrector. We also intend to use information extraction techniques to extract additional information about the locations of the infected host often mentioned in the unstructured texts of the *notes* and *comments* metadata fields. Our future work would also include exploring additional resources containing species information, such as Interagency Taxonomic Information System (72), Encyclopedia of Life (73) and Catalogue of Life (74), for host name normalization instead of relying solely on the NCBI Taxonomy database, which has missing entries for the alternative names of some organisms. In addition, we intend to modify our host normalization algorithm so that it is capable of recognizing varying degrees of taxonomy hierarchy, thereby allowing normalization to different levels of the taxonomy tree based on user needs. Addition of this feature would facilitate the normalization of host names which cannot be mapped to a single organism, since they could instead be mapped to higher nodes in the taxonomy tree, and would be highly useful in case of viruses which may live in different host organisms. Further important steps we plan to take through future work include developing normalization algorithms for other metadata in GenBank and extending our normalization algorithms to other non-virus organisms (72).

## Funding

National Library of Medicine (NLM) of the National Institutes of Health (NIH) (R01LM012080) to M.S.; National Institute of Allergy and Infectious Diseases (NIAID) of the NIH (R01AI117011) to M.S. and G.G. The content is solely the responsibility of the authors and does not necessarily represent the official views of the NIH.

*Conflict of interest*. None declared.
